# A qualitative study of Ottawa university students’ awareness, knowledge and perceptions of infertility, infertility risk factors and assisted reproductive technologies (ART)

**DOI:** 10.1186/1742-4755-10-41

**Published:** 2013-08-20

**Authors:** Kelley-Anne Sabarre, Zainab Khan, Amanda N Whitten, Olivia Remes, Karen P Phillips

**Affiliations:** 1 Interdisciplinary School of Health Sciences, Faculty of Health Sciences, University of Ottawa, Ottawa, ON, Canada; 2 Institute of Population Health, University of Ottawa, Ottawa, ON, Canada

**Keywords:** Infertility, Qualitative, University students, Risk perception, ART

## Abstract

**Background:**

Awareness of infertility risk factors is an essential first step to safeguard future fertility. Whereas several studies have examined university students’ awareness of female fertility and related risk factors, the topic of male infertility has not been well examined. The objective of this study was to assess young men and women’s awareness, knowledge and perceptions of infertility, male and female infertility risk factors and assisted reproductive technologies (ART).

**Methods:**

Semi-structured interviews were conducted in 2008 with a multi-ethnic sample of sixteen male and twenty-three female Ottawa university students, followed by qualitative data analysis to identify major themes. Interview topics included awareness of male and female infertility risk factors, infertility diagnosis/treatments and personal options in the event of future infertility.

**Results:**

Participants were generally familiar with infertility as a biomedical health problem, could identify sex-specific risk factors but overestimated fertility of women in their thirties and ART success rates. Reproductive health knowledge gaps and confusion of the physiological life-stage of menopause with infertility were apparent. Most participants would pursue in vitro fertilization or international adoption in the event of personal infertility. Some participants wished to use a ‘natural’ approach and were concerned with potential side effects of ART-related medications.

**Conclusions:**

The general awareness of infertility in young adults is promising and supports the potential uptake for health promotion of fertility preservation. This study underscores the continued need for comprehensive sexual and reproductive health education and promotion for adolescents and young adults.

## Introduction

Infertility is the inability to conceive children after one year of unprotected intercourse [1]. The estimated prevalence of infertility in Canada is 11.5-15.7% [2]. The risk of infertility increases with advanced age of the female partner (>35 years) [1-4]. Female infertility may present as anovulation, obstructed fallopian tubes, endometriosis or uterine abnormalities [1,3]. Male factor infertility is characterized by diminished production of morphologically normal, motile sperm [3,5]. Genetic abnormalities, hormonal imbalances and congenital/infectious malformations of the reproductive tract are some of the common causes of male and female infertility [1,3,5,6]. Lifestyle factors such as obesity [7], diet, smoking and alcohol use [8] along with environmental chemical exposures [1,5,8] have been increasingly examined as additional modifiers of fertility.

Diagnosis of infertility is varied and may include assessment of sperm quality, hormones and imaging analysis of the uterus/fallopian tubes. Dependent on the diagnosis, infertility may be treated by reproductive surgery, administration of hormones and/or assisted reproductive technologies (ART). ART encompass clinical/laboratory procedures wherein male and female gametes are manipulated for the purposes of reproduction and include in vitro fertilization (IVF), intracytoplasmic sperm injection, preimplantation genetic diagnosis, embryo cryopreservation and gestational surrogacy [9].

Infertility awareness, including knowledge of male and female risk factors, is a critical first step towards fertility preservation through lifestyle modification [1,4]. Rather than personal fertility or parenting experience, fertility knowledge is instead associated with education [10]. Health promotion strategies are therefore recommended to begin with educational interventions [10]. Fertility health promotion represents an important gap in the literature. In Ontario, there is little to no mention of infertility in compulsory secondary school sexual health curricula [11], an important source of sexual and reproductive health (SRH) information for Ontario-matriculated university students [12,13]. It is well established that awareness of infertility risk factors is essential for fertility preservation [4,8,10,14], however there exist important gaps in such reproductive health promotion in North America [15].

The discourse around ART, infertility and associated ethical, legal and regulatory issues has permeated Western culture. Population studies confirm that Canadians receive health information most frequently from medical doctors along with news media and the Internet [16]. Although media has framed infertility/ART as mainstream cultural phenomena [17], this does not necessarily indicate a general awareness of infertility risk factors and strategies for fertility preservation. Previous studies of Swedish [18-20], Finnish [21], Italian [22], American [23], United Kingdom (UK) [24], Israeli [25] and Canadian [26] university students have reported gaps in awareness of female infertility risk factors and an overestimation of the fertility of women in their late thirties. Infertility does not always originate in the female, with male factor infertility contributing to about half of all cases [3]. It is therefore essential for young men to also be aware of sex-specific infertility risk factors along with diagnosis and treatment options. We found few male infertility risk perception studies among them studies of adult Australians (mean age 27.3 years) [27], childless Canadian men (mean age 33.9 years) [28] and Toronto, Canada high school students (mean age 17.5 years) [29]. Whereas the Australian sample included mature adults of which 30% were parents [27], the Toronto cohort represented a life-stage transitioning through adolescence [29] and not in contemplation of future parenthood [12,13]. University years are a time for self-maturation which includes sexual exploration and pregnancy avoidance during this life-course transition to adulthood [30]. Several studies have reported that university students place a significant emphasis on financial/career stability and degree completion as prerequisites to parenthood [31,32]. Here, we have designed a qualitative study to more broadly examine university students’ awareness, perceptions and knowledge of both male and female infertility risk factors, infertility diagnosis, treatments and options in the event of personal infertility.

## Methods

### Participants

Undergraduate students were recruited from a large, ethnically diverse university in Ottawa, Ontario, Canada using posters, snowball technique and FaceBook advertisements. The recruitment text invited students to participate in one-on-one semi-structured interviews to discuss their knowledge and understanding of issues pertaining to infertility. Students were reimbursed with a $10 coffee card for participation time (~1 hour). Ethics approval was granted by the institutional ethics review board and all respondents provided written consent to participate. A multiethnic sample of sixteen young men (mean age 20.4 years) and twenty-three women (mean age 21.6 years; Table 1), childless with the exception of one male, participated in this study. The mean age of the entire sample was 21 years. Twenty-nine participants intended to have children in the future. This sample can be considered academically privileged with access to on campus health promotion and educational resources.

**Table 1:** Participant characteristics

**Characteristic**	**Value**	**Definitely want children**^**1**^
**Gender**		
female	23	15
male	16	14^2^
**Age**		
female age range	19-26	19-26
(mean ± SD) years	(21.6 ± 1.9)	(21.9 ± 2.1)
male age range	19-24	19-24
(mean ± SD) years	(20.4 ± 1.5)	(20.4 ± 1.6)
**Ethnic/Cultural Identity**^**3**^		
Canadian	19	15
African	2	2
Asian	8	5
European	4	3
Middle Eastern	6	4

^1^A total of ten participants did not identify a strong desire to have children: eight participants indicated they do not plan to have children (6 females, 2 males); two female participants were unsure.

^2^One Canadian male participant, aged 24, indicated he had one child at the time of the study and would like to have additional children.

^3^Ethnic Categories as follows: **African** (Ethiopian), **Asian** (Chinese, Hong Kong, Indian, Indonesian, Korean, Pakistani, Philippine); **European** (Finnish, British, Bulgarian, Irish, Italian), **Canadian** (African Canadian, Algerian-Canadian, British Canadian, Canadian, French Canadian, Hong Kong-Canadian, Persian-Canadian, Sikh-Canadian), **Middle Eastern** (Iranian, Kuwait, two participants provided “Middle-Eastern”, “Middle-Eastern/Arab” as their stated identity).

SD- standard deviation.

### Data collection

Thirty-nine individual, semi-structured interviews were conducted January to December 2008 and audio-recorded/noted. The interview guide was broadly designed by the research team to query participants’ awareness and perceptions of biological, environmental and social aspects of infertility and included open-ended, yes/no and structured response questions. Some questions were modified from previously published quantitative studies [18,19]. Here we present the portion of the study exploring participants’ awareness, knowledge and perceptions of infertility diagnosis, sex-specific risk factors, treatments and personal options in the event of future infertility (Table 2). Demographic data including age, race/ethnicity, parenthood status and desire for children were also collected.

**Table 2:** Interview Questions

**Topic**	**Questions**
Infertility: definition, diagnosis	Define infertility
	How is infertility diagnosed?
Causes of infertility	What are some of the causes of infertility among women? What is the biggest cause?
	What are some of the causes of infertility among men?
	Does age affect infertility for men? YES/NO
	Does age affect infertility for women? YES/NO
	At what age is there a marked decrease in women’s ability to become pregnant? Please select from: 25–34; 35–39; 40–44; 45+; no association between age and ability to become pregnant
Infertility treatment: options, success	What options are available for those who are infertile?
	For couples that undergo treatment with in vitro fertilization, how likely are they of having a child?
	Can infertility be cured?
In the event of personal infertility…	If you were infertile, which of the following options - adoption, surgery IVF, medication/pills or not having children -would you consider? What factors made you choose [selected infertility options]?

### Analysis

Transcription of the audio-recorded interviews was followed by exploration of the data using qualitative content analysis [33]. Briefly, data analysis was organized by interview topic with frequency of responses used to construct content themes or codes. Transcripts were coded using NVIVO™ (QSR International, Cambridge, MA, USA) by three health sciences undergraduate students with major themes emerging inductively as agreed upon by the research team through consensus. A sex-based analysis enabled comparisons of men and women’s response to the interview topics. Periodic research team coding enabled refinement of the coding grid, theme categorization and confirmation of saturation [34,35]. Some interview questions (Table 2) elicited either YES/NO responses or required participants to select responses from a list. For these questions, frequencies of responses were determined and reported in the text.

## Results

Major themes and subthemes organized by interview topic appear in Table 3.

**Table 3:** Major themes and subthemes organized by interview topic

**Interview topic**	**Major theme(s)**	**Subtheme(s)**
Define infertility.	Inability to have children	Infertility associated with underlying biological/medical problem (women)
How is infertility diagnosed?	Diagnosed by doctor	Medical tests
	Trying and not conceiving	
Causes of female infertility?	Advanced maternal age	Lifestyle, Genetics
Causes of male infertility?	Lifestyle (men)	Genetics (men), Advanced paternal age (men)
	Low sperm count (women)	Lifestyle (women)
Infertility options?	IVF	Adoption
		Artificial insemination (men)
		Surrogacy
Chance that IVF will produce a child	Moderate-high chance success	Popularity of IVF
		Skill of physician
		Expensive, uncertain, arduous
Infertility curable?	Infertility curable (men)	Promise of technology
		Future developments
Options in event of personal infertility	Adoption	Natural approach
	IVF	Concerns about medication side effects
	Childlessness (men)	

### Infertility: definition, diagnosis

Participants were asked to define infertility which provided a baseline for the future infertility-related questions. All participants had a general understanding of what is meant by the term infertility, evidenced by the major theme: *the inability to have children* (18/23 women, 14/16 men)*.*

*From what I know, infertility is like…after married and then you had sex and everything and you’ve been trying and trying for a long time and you still can’t have kids.* ID-11; male, aged 21, Indonesian

*That you can't produce children even though you and your partner have regular sexual intercourse* ID-31; female, aged 22, Iranian

The association of infertility with an *underlying biological/medical problem* emerged as a subtheme for women (6/23).

*Infertility it’s when you’re unable to have a child because of either different medical reasons and maybe some conditions that you were born with or maybe something in the environment that causes you to become infertile later in life.* ID-3; female, aged 22, Algerian-Canadian

[Infertility] *this just means that you cannot produce enough eggs or sperm to have children* ID*-*27; female, aged 21, French-Canadian

Participants were not confident describing mechanisms of infertility diagnosis, with most providing multiple responses. Major themes included: *diagnosed by doctor* and *trying and not conceiving.* Participants commonly indicated that infertility was ultimately confirmed by a doctor (14/23 women, 7/16 men) through a *series of medical tests* (subtheme).

*A physical check-up. If you try to have children and you can’t. And then he would, or she would do a whole bunch of tests.* ID-16; female, aged 19, Canadian

*If, I guess, when you can’t conceive a child then you might wanna go see a doctor, see if there’s a problem with your reproductive organs Because they can do a series of tests, I’m not really sure what they might do, but with some medical and diagnostic tests.* ID-18; female, aged 22, Persian-Canadian

Several women (11/23) and men (4/16) explained infertility diagnosis as *trying and not conceiving*; recognizing that prior to medical intervention the couple must attempt conception through regular sexual intercourse.

*Yeah like if you tried for a significant amount of time and you just found that it wasn’t happening then obviously I’d probably end up going to the doctor telling them we’ve been trying for a couple of months and nothing’s been happening.* ID-20; female, aged 20, Sikh-Canadian

*You keep having sex and trying but the lady isn't getting pregnant* ID-28; male, aged 22, Ethiopian

Many participants recognized that medical tests would be *sex-specific* suggesting separate, yet unspecified, tests for males or females. Although some participants were able to identify semen analysis as part of male infertility investigations (6/23 women, 4/16 men), both men and women were less familiar with female infertility assessments. Only two men and three women identified medical testing of eggs, ovulation or hormones in the investigation of female infertility.

*Well depends on the person who’s infertile, is it the woman or is it the male? Don’t they test your sperm and see if the sperm cells are fertile or not? Or if they check the egg and see if it’s a problem with the eggs or something.* ID-5; male, aged 19, Kuwaiti

*I think it depends on the condition that you have, for some it could be that they lose their ovaries as a result of cysts. For men, they don’t have a sperm count. For women, it could be hormone levels. I think it really depends on the condition that you have and the stage of the disease or illness or whatever you wanna call it, condition. Through a series of tests, obviously there is ultrasound tests that they could do. I don’t know if blood tests would work, but I don’t think so. Well, for men they could do sperm counts.* ID-10; female, aged 22, Canadian

*At infertility clinics, I don’t know. I’m sure they test on sperm count and motility of sperm and your egg production.* ID-14; female, aged 21, Canadian

In general, both men and women expressed a good understanding of the definition of infertility and the need for medical diagnosis. Knowledge gaps included lack of awareness of specific diagnostic tests for women or early warning signs such as irregular menstrual periods, anovulation, excessive bleeding or pain that might indicate fertility problems. No race/ethnicity-specific patterns were apparent.

### Causes of infertility

#### ***Identified causes of female infertility***

Men and women provided multiple causes for female infertility. *Advanced maternal age* was the major theme describe by women (12/23) and men (8/16). Subthemes included *lifestyle* (substance abuse/diet/smoking/stress; 5/23 women, 4/16 men) and *genetics* (4/16 men; 4/23 women).

*Age. Genetics, if there is a family history for it then they'd be affected too I think.* ID-21; male aged 20, Chinese

*I guess genetic disorders, like say you have a chromosomal disorder where you don’t have a, I think for women it’s XX right? And then if you have an extra sex chromosome then it can, then you’ll be infertile and you can’t have children.* ID-18; female, aged 22, Persian-Canadian

*Age and I imagine illnesses such as cancer and other chronic diseases like obesity and diabetes.* ID-27; female, aged 21, French-Canadian

When asked to identify the biggest cause of female infertility about half of men (9/16) and women (13/23) identified *advanced maternal age*. Given a list of age ranges, most male and about half of the female participants indicated that women’s fertility decreased significantly only after age 40, representing a critical knowledge gap. The correct age range (35–39) [4,36] was identified by only five women and two men. When asked specifically whether age contributed to female infertility almost all participants (22/23 women, 15/16 men) responded affirmatively, with no apparent race/ethnicity or gender-specific trends.

*I’ve heard 35 is a tough barrier to break for a woman.* ID-2; male, aged 20, Canadian

*Their eggs just get worse over time.* ID-5; male, aged 19, Kuwaiti

*Cause you can’t have kids past 40, 45, when you go through menopause*, ID-10, female, aged 22, Canadian

*Yes. For women, as you get older, I think your estrogen levels go down, I think. And then there’s obviously menopause.* ID-18; female, aged 22, Persian-Canadian

Infertility and reproductive health knowledge gaps were sometimes apparent. Most participants could articulate a general definition of infertility but a few lacked reproductive anatomy/physiology knowledge and many confused menopause with infertility. Responses often included *‘I’m not sure’,* ‘*I think so’* or *‘I guess’,* suggesting participants were less certain about infertility risk factors.

*Some women aren’t even born with eggs, so that’s infertility right there.* ID-6; female, aged 21, Canadian

*As far as I know, I think all women go through menopause right?* ID-19; male, aged 19, Ethiopian

#### ***Identified causes of male infertility***

*Lifestyle* (drinking/alcohol/smoking/stress; 7/16) emerged as the major theme for men’s perceptions of risk factors for male infertility followed by subthemes *genetics* (4/16) and *advanced paternal age* (4/16) In contrast, women predominantly identified *low sperm count* (8/23), followed by *lifestyle factors* (6/23).

*A lot of people who have drug problems have a low sperm count, that also affects their sex drive, their libido, so that makes them more likely to be infertile.* ID-6; female, aged 21, Canadian

*Probably drug abuse, diet, alcohol, age probably too. ID-9;* male, aged 20, Chinese

When asked specifically about risks of paternal age to fertility a gender gap emerged. Men (11/16) identified advanced paternal age as a risk factor for infertility whereas women (14/23) did not believe paternal age was a significant infertility risk. Women considered paternal age relative to the greater infertility risk of advanced maternal age.

*The popular notion is that* [age] *doesn’t, that men can have children until they’re old-aged, but I think as you age your body kind of breaks down a little bit and so that probably would impact men as well. Maybe not to as great of an extent as women. I would say yes.* ID-3; female, aged 22, Algerian-Canadian

*It’s not supposed to, but it probably…the number of sperm could actually, probably decrease but there should still be some fertile sperm. Yeah I said that reproduction will decrease but the sperm, I’m thinking, should still be fine. They’re created all the time. But women, yes.* ID-13; female, aged 22, Canadian

[Men] *can have babies until they die.* ID-33; female, aged 21, Arab-Middle Eastern

Knowledge gaps were evident with eight women and two men, identifying ‘low sperm count’ as a cause rather than correctly identifying it as a manifestation/sign of infertility. Only one man and two women were unable to identify any causes for male infertility, with confusion of anatomy/physiology sometimes apparent.

*I never knew men could be infertile to be honest, I guess malfunction of the penis. Being unable to I guess deliver the sperm to where it needs to go.* ID-17; male, aged 19, Kuwaiti

*I don’t know as much about infertility among men but maybe some accidents, like some things in the environment... If there’s damage to that anatomical part that’s caused by external things and also obviously maybe prostate cancer.* ID-3; female, aged 22, Canadian

### Infertility treatment: options, success

Both male and female participants identified multiple infertility treatment options. The major theme to emerge was *IVF* (14/23 women, 8/16 men) followed by sex-specific subthemes *artificial insemination* (4/16 men) and *surrogacy* (4/23 women, 3/16 men). *Adoption* was a dominant subtheme for both male (5/16) and female participants (13/23) and was perceived as an option in the event of infertility.

*IVF seems to be popular these days.* ID-26; female, aged 23, Indian

*There’s artificial insemination, there’s hormones. In a lab, they get an egg and sperm. Yeah, they implant it into the uterus* ID-9; male, aged 20, Chinese

*Adoption, IVF, have someone else carry your baby for you… I forget what that is called.* ID-30, female, aged 21, Irish

Women (14/23) and men (12/16) perceived IVF to have a ‘moderate’ to ‘high’ chance of success of producing a child based on its popularity and the skill of the physician.

*I think on the most part it works, because a lot of people see it as an option. Don’t really know. I would assume so*. ID-6, female, aged 21, Canadian

*I think their chances are better just because it’s done by the doctors and the scientists when they put it together so it’s been proven to work many times - the success rate has been good so it’s more likely that it’ll work.* ID-5, male, aged 19, Kuwaiti

*Pretty high, maybe 80%.* ID-27, female, aged 21, French-Canadian

Other respondents were aware that the process of conceiving a child through IVF can be arduous, uncertain and expensive.

*I’d say be prepared that you just spent your money and it didn’t quite work.* ID-2; male, aged 20, Canadian

*I’d say unlikely. Yeah. Only from what I see with my own friends, then again, they only stop because of financial reasons. Like they might go three times, that’s like $20,000 every time, so that’s about all they give.* ID-13, female, aged 22, Canadian

*It works but I know that you have to try several times because the eggs are very sensitive and sometimes they can’t fertilize it properly. Sometimes when they insert the egg into the uterus then it might not work.* ID-18, female, aged 22, Persian-Canadian

Men (10/16) were more likely than women (5/23) to perceive infertility as curable. Subthemes included *the promise of technology* and *future developments.*

*Yes with all the new technology it should be.* ID-28; male, aged 22, Ethiopian

*Yeah, I think it’s possible. I don’t know, they’ve come pretty far with medicine, so I would presume there’s a lot of things they can do, especially with the new stem cell research and stuff.* ID-8; male, aged 19, Chinese-Canadian

*In the future probably. Now? No I don’t think so, there’s not enough known about it to cure everyone.* ID-14, female, aged 21, Canadian

Almost all participants were familiar with IVF and were aware that infertility could be treated. Some participants expressed general awareness of the expense of infertility treatments and associated emotional stress. Important knowledge gaps included overestimation of the success rates of IVF and the ease/access of adoption.

### In the event of personal infertility

Participants who expressed a strong desire to have children predominantly chose adoption (13/23 women, 12/16 men) followed by IVF (9/23 women, 10/16 men) as options for future infertility, with more men (7/16) settling for childlessness than women (4/23). Participants emphasized choices that were *natural* and expressed concerns about *medication side effects*.

*Adopting and in vitro fertilization. Because the in vitro one, it seems like it has a very good success rate so there’s a pretty good chance that we’d have a baby. And adopting, if I wouldn’t be able to have child then I would want to adopt a child, make their life better – improve it, take them in.* ID-5, male, aged 19, Kuwaiti

*Adopting. Well I’ve kind of always wanted to adopt whether or not I was fertile or I would be infertile. And any of the other reasons, I really haven’t ..feel comfortable with some kind of scientific. Probably the actual procedure. I just feels, well I mean it is less natural.* ID-19, male, aged 19, Ethiopian

*No to surgery because that’s scary, IVF- I don't know what this is. No to medication and pills because they have side effects, I would want to adopt.* ID-31, female, aged 22, Iranian

Choices in the event of personal infertility did not exhibit race/ethnicity patterns; instead treatment safety concerns, a desire for a ‘natural’ approach and the altruism of adoption seemed to influence participants’ selections.

## Discussion

Ottawa undergraduate students expressed a general understanding of infertility, sex-specific risk factors and the need for medical diagnosis of infertility. Infertility was framed as a biomedical health problem, disease or dysfunction of the reproductive system. In contrast, only a small proportion of American, European and Australian adults defined infertility as a disease [37]. This may reflect the academic setting of our study, as education is positively correlated with fertility knowledge [10]. Health promotion strategies for cancer risk prevention [38] and STI risk reduction [13,39,40] illustrate that awareness of disease risk factors is a prerequisite to positive health behavior change. Male participants readily identified lifestyle risks (substance abuse, smoking, alcohol) to male infertility; consistent with the responses of childless Canadian men [28] and Toronto high school boys [29]. In contrast, only a third of Australian men identified smoking as a risk factor for male infertility [27] which may reflect regional differences in health promotion or health education. Women, and to a lesser extent men, identified low sperm count as another risk to male infertility. This suggests participants’ confusion between manifestations of male infertility and the antecedents of impaired semen quality. Obesity, a well-established risk factor for male infertility [7], was not identified by our participants in contrast to about a third of Australians [27] and Toronto high school boys [29]. Occupational exposures, including radiation, environmental contaminants and temperature extremes [5,41], were mentioned by only two participants as risks for male infertility, reflecting an important knowledge gap. Health promotion targeting men should include identification of modifiable risk factors to male infertility including lifestyle, obesity and occupational exposures.

Participants expressed mixed perceptions about the fertility risks of advanced paternal age, consistent with the Australian [27] and Canadian [28,42] studies and supported by the reproductive health literature [43]. Although some laboratory studies [44,45] report an association of advanced paternal age and reduced semen quality, other groups found no age effect [46-48]. Similarly, clinical studies both confirm [49,50] and refute [51,52] associations between advanced paternal age and reduced live birth rates. As it seems likely that there is some association between advanced paternal age, infertility and adverse fetal outcomes [53], promotion of fertility preservation should address this risk issue.

The literature is not equivocal about the risk of maternal age to infertility [2-4,36]. Participants demonstrated strong awareness of female age as a risk factor for infertility, but were less able to identify the age at which fertility begins to decline. Most of our male participants and about half of our female participants indicated female fertility decreases only after age 40. The lack of awareness of the age 35 ‘threshold’ [2,3,54,55] could have important implications for future family planning. These findings are consistent with several studies of European [18-22], American [23], UK [24], Israeli [25], and Canadian [26] university students, and Australian [27] adults. In contrast, studies of childless Canadian adults report recognition of the decline in female fertility after age 35 years; however these men and women also consider overall health and fitness of women over age 30 years to be more important predictors of fertility than age [28,42]. Participants were somewhat confused about physiological life-stages, with a tendency to associate this decline in female fertility with onset of menopause; representing another important knowledge gap.

In addition to advanced maternal age, participants identified a range of infertility risk factors for women including lifestyle and genetics. Although our sample by age is in the highest STI risk group (individuals under the age of 30) [56] most participants did not identify STI as risk factors for female infertility; consistent with the Toronto high school sample [29]. Most Canadian adults, however, do recognize that STI such as gonorrhea and chlamydia can contribute to female infertility [28,42]. Although the compulsory Ontario secondary school sexual health curriculum [11] places a strong emphasis on STI risk reduction, our previous work suggests that Ottawa secondary school sexual health teachers rarely discuss associations between STI and infertility [13]. STI are well-established risk factors for female infertility [6], with evidence somewhat unclear for male infertility [57]. Long term consequences of STI, including infertility, may represent another gap in reproductive health promotion for this young adult population [12,13].

Most participants recognized that infertility investigations required a period of trying unsuccessfully to conceive, followed by sex-specific diagnostic tests. Although semen assessment was identified as a diagnostic test for male infertility, few participants could discuss investigations for female infertility. None of the participants discussed changes in menstrual period regularity/pain or other symptoms which could be recognized by women as potential infertility warning signs. A UK reproductive health promotion strategy FertiSTAT has been developed as a fertility awareness tool for women and includes menstrual cycle irregularity, menstrual pain and history of STI among risk factors for female infertility [58]. Such fertility health promotion tools have obvious benefit for personal risk assessment.

IVF, artificial insemination and surrogacy were identified as infertility treatments, with the success of IVF greatly overestimated by Ottawa university students. Similarly, over 90% of childless Canadian adults perceive that ART can help most premenopausal women conceive [28,42]. Most male and several female participants believed that medicine/science could ‘cure’ infertility; consistent with similar studies [18,19,29]. There is a clear discordance between North American ART live birth rates (25-35% for most clinics) [59,60] and participants’ perceptions of ART efficacy. Participants’ perceived ‘faith’ in the promise of medical technologies is consistent with a risk perception study which reported that Canadians place significant confidence in doctors and university scientists for health information [16]. This may also explain why even with very little understanding of the process and efficacy of IVF most participants who desired to have children in the future would consider use of IVF in the event of personal infertility. A study of childless Canadian adults reported both men and women were open to using IVF but were less willing to consider third-party options (i.e. donor eggs/embryos, surrogacy) [61]. ART uptake has increased over the past decade in Canada [59] and the US [60], supporting the need for health promotion regarding fertility preservation and the risks and benefits of ART.

Reproductive decision making and perceptions of infertility can be influenced by religious and cultural beliefs [62,63], however ethnic-minority participants responded similarly to those identifying as ‘Canadian’, with no mention of family, tradition or cultural influence on their personal choices in the event of infertility. This may reflect the structure of our interview questions which emphasized infertility definitions, risk factors and treatment options. Further, all participants, regardless of ethnic/cultural identity or gender, would have some general SRH knowledge from local media, Internet and on-campus health promotion which may explain the relative homogeneity of responses.

Both men and women in our sample were strongly attracted to adoption as a solution to personal infertility. Adoption may have been perceived as a popular alternative to ART given some participants’ concerns regarding safety and a desire for the most ‘natural’ approach. This is consistent with perceptions of childless Canadian adults, most of whom perceive IVF as a health risk for women [28,42]. Overwhelmingly adoption was discussed positively, as a welcome family planning option and as an altruistic act. Foreign, but not domestic, adoptions were explicitly mentioned. Although expense was identified as a concern with IVF, participants did not seem to recognize the financial expenses related to adoption. Further, participants seemingly overestimated the ease of transnational adoption with no discussion of complex social, ethical and legal barriers [64].

### Limitations

A qualitative study design was a valuable methodological tool to assess knowledge, awareness and perceptions related to infertility, however there were limitations. Methodologically, we chose a semi-structured interview approach to elicit participants’ awareness of infertility facts. This approach may have prevented participants from exploring their personal perceptions, experiences with pregnancy prevention, pregnancy or abortion during the interviews. Our complex semi-structured data-set was primarily assessed using a simple qualitative content analysis, with frequency analysis used for closed-ended questions. A theoretical-designed interview guide comprised solely of open-ended questions would have required a more rigorous qualitative analysis, yielding a richer data-set regarding the antecedents of the participants’ infertility perceptions such as education, culture and experience. We recruited a multi-ethnic sample typical of the study setting and consistent with Canada’s multicultural makeup. This strategy produced a heterogeneous sample with multiple regional representations, which in turn impaired our ability to perform cross-cultural analysis. Although we did not collect citizenship data, approximately 95% of undergraduate students from the host university are Canadian/permanent residents [65] with 65.8% entering university directly from an Ontario secondary school [66]. Ethnic/cultural identities other than Canadian do not necessarily preclude Canadian citizenship/permanent residency. As university students, our respondents represent a privileged group in terms of education and access to on-campus health promotion. A cohort of age-matched young adults without post-secondary school education may have different perceptions and awareness of infertility. Another limitation of this study was the exclusion of data collection related to sexual identities. Participants’ family planning desires may have been informed by their sexual identity as heterosexual, gay, lesbian, bisexual, transgender or questioning (LGBTQ). ART access and related infertility support services exist for LGBTQ Canadians [67], although few studies have examined this issue.

## Conclusions

This sample of young adults is generally open to infertility treatment options but overestimates the success of ART and transnational adoption access. Both men and women in our study were able to describe infertility as a biomedical health problem and could identify multiple risk factors for male and female infertility. There remain however, significant knowledge gaps and confusion regarding the physiological age-dependent decline in female fertility and the role of STI as infertility risk factors. A basic understanding of the male and female reproductive systems, sexual function, puberty, pregnancy and menopause, is essential to help individuals negotiate healthy sexual relationships, navigate developmental life stages and ensure reproductive health and fertility. Our results provide support for continued SRH promotion with expanded emphasis on fertility preservation.

## Competing interests

The authors declare that they have no competing interests.

## Authors’ contributions

ZK and OR performed interviews, K-AS, ANW and OR contributed to data analysis, KPP designed study, directed co-authors’ research activities, designed and provided oversight to data analysis, drafted manuscript. All authors reviewed, edited and approved the final manuscript.
